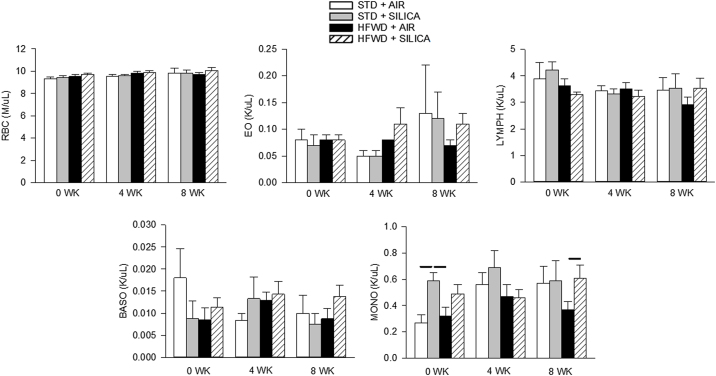# High-fat western diet-consumption alters crystalline silica-induced serum adipokines, inflammatory cytokines and arterial blood flow in the F344 rat

**DOI:** 10.1016/j.toxrep.2021.12.001

**Published:** 2021-12-07

**Authors:** Janet A. Thompson, Kristine Krajnak, Richard A. Johnston, Michael L. Kashon, Walter McKinney, Jeffrey S. Fedan

**Affiliations:** Health Effects Laboratory Division, National Institute for Occupational Safety and Health, Morgantown, WV 26505, United States

**Keywords:** Obesity, Silicosis, Adipokines, Inflammation, Cytokines, Adipose tissue

## Abstract

Adipose tissue (AT) plays a central role in the maintenance of whole-body energy homeostasis through release of adipokines. High-fat Western diet (HFWD)-consumption contributes to obesity, disruption of adipocyte metabolism, chronic systemic inflammation, and metabolic dysfunction (MetDys). MetDys is associated with impaired lung function, pulmonary hypertension, and asthma. Thirty-five percent of adults in the U.S. have MetDys, yet the impact of MetDys on susceptibility to occupational hazards is unknown. The aim of this study was to determine the potential of HFWD-consumption to alter inhaled crystalline silica dust-induced metabolic responses. Six-wk old male F344 rats were fed a HFWD (45 kcal % fat, sucrose 22.2 % by weight) or standard rat chow (STD, controls), and exposed to silica-inhalation (6 h/d, 5 d/wk, 39 d; Min-U-Sil 5®, 15 mg/m^3^) or filtered air. Indices of MetDys and systemic inflammation were measured at 0, 4, and 8 wk following cessation of silica exposure. At 8 wk post-exposure, silica reduced serum leptin and adiponectin levels, and increased arterial pulse frequency. HFWD-consumption induced weight gain, altered adipokines, liver, kidney, and pancreatic function, and increased tail artery blood flow. At 8 wk in HFWD + SIL-treated animals, the levels of serum pro-inflammatory cytokines (IFN-γ, CXCL-1, TNF-α, IL-1β, IL-4, IL-5, IL-6, IL-10 and IL-13) were increased compared to STD + SIL but were less than HFWD + AIR-induced levels. In conclusion, consumption of a HFWD altered silica-induced metabolic responses and silica exposure disrupted AT endocrine function. These findings demonstrate previously unknown interactions between HFWD-consumption and occupational silica exposure.

## Highlights

• Silica reduced serum leptin and adiponectin, no effects on body or fat pad weight.
• HFWD-consumption altered pro-inflammatory cytokines in silica-exposed animals.
• Silica altered pulse frequency; HFWD increased mean blood flow; effects additive.
• HFWD affected silica-induced metabolic effects.

## 1 Introduction

Adipose tissue (AT) is involved in energy storage and uptake of triglycerides from the circulation. AT expresses and secretes cytokines known as adipokines, e.g., leptin and adiponectin. Leptin is involved in the satiety response, which is mediated through its hypothalamic receptors [1], and adiponectin contributes to maintenance of insulin sensitivity of cells [2]. In obesity, the uptake and storage of excessive amounts of circulating triglycerides induces adipocyte stress. Stressed adipocytes exhibit altered endocrine function, e.g., increased leptin and decreased adiponectin secretion, and release inflammatory mediators and undergo apoptosis due to activation of the NLRP3 inflammasome [3,4]. In turn, AT macrophages become activated, release pro-inflammatory cytokines and mediators which recruit and activate additional immune cells, undergo NLRP3 inflammasome activation and apoptosis, and contribute to a cycle of non-resolving inflammation in the AT [5]. As local adipose inflammation increases, cytokines are released into the circulation, creating chronic, low-grade systemic inflammation. The combination of altered adipokine levels and systemic inflammation contribute to the development of insulin resistance, atherosclerosis, dyslipidemia, hypertension, and other conditions known as metabolic dysfunction (MetDys) [6,7,4]. Thirty-five percent of adults in the U.S. have MetDys [8].

Consumption of a HFWD, obesity and associated MetDys may influence a worker’s susceptibility to occupational inhalation hazards. MetDys biomarkers, i.e., elevated triglycerides, heart rate and leptin and low HDL, were identified as risk factors for susceptibility to severe lung function impairment in first responders following the 9/11 World Trade Center attack [[9], [10], [11], [12]]. Coal miners diagnosed with silicosis and MetDys are at increased risk for atherosclerosis [13]. Appalachian coal miners are more likely to be overweight (87 %), obese (52 %), or have hypertension (31 %), compared to the general population [14,15] and at the same time silicosis cases in these miners have steadily increased [14]. Animal studies suggest that HFWD-consumption combined with occupational exposures can result in altered metabolic function [16]. Together, these studies indicate that HFWD-consumption and associated MetDys may intensify responses to occupational inhalation hazards.

Approximately 1.2 million American workers in industries including construction, mining, and stone cutting are exposed to inhaled crystalline silica and are at risk for developing silicosis [17,18], an irreversible inflammatory and fibrotic lung disease. Respirable-size silica particles deposited in the alveoli are phagocytosed by alveolar macrophages (AMs) and activate the NLRP3 inflammasome. This stimulates AMs to release reactive oxygen species (ROS), reactive nitrogen species (RNS), free radicals, inflammatory cytokines, proteases, leukotrienes, chemokines, and chemoattractant proteins [[19], [20], [21], [22], [23]]. Silica particles transported intracellularly to the AM lysosome disrupt the lysosomal membrane, causing release of lysosomal contents into the cytoplasm, leading to apoptosis and release of the particle into the alveolar space. Factors released by the AMs, including TNF-α, IL-1β and TGF- β, promote alveolar fibroblast collagen and elastin deposition, which result in the development of pulmonary fibrosis. A cycle of reuptake and release of silica particles, AM cell death, and inflammation and fibrosis in the alveoli, all contribute to the progression of silicosis [[24], [25], [26], [27]].

For this study we used a well-characterized F344 rat model of silicosis [24,26,[28], [29], [30]] to mimic occupational crystalline silica-inhalation exposure. F344 rats continually exposed to respirable crystalline silica (15 mg/m^3^ silica, 6 h/day, 5 d/wk) develop a biphasic pulmonary inflammation response. The initial response consists of a significant but steadily maintained state of pulmonary inflammation for the first 40 days, after which there is an explosive inflammatory lung response which continues to increase with time [24]. Porter et al. [29] demonstrated that a silica particle threshold exists and, once exceeded, despite cessation of silica exposure, pulmonary inflammation will continue, and development of fibrosis will ensue. This animal model best mimics the accelerated type of silicosis in which workers exposed to a large amount of silica over a short period of time develop progressive pulmonary inflammation and fibrosis that continues even after silica exposure has ended [20].

This study investigates the impact of HFWD-consumption on occupational crystalline silica inhalation-induced metabolic effects. While the detrimental effects of HFWD-consumption in humans are well documented, the impact of HFWD-consumption in conjunction with hazardous occupational exposures are widely unknown. Here we examine various indices to determine whether HFWD-consumption alters metabolic responses in silica-exposed animals. To our knowledge, there are no previous studies examining HFWD-consumption and silica inhalation-induced metabolic interactions.

## 2 Materials and methods

A complete description of methods used are included in the supplemental data.

### 2.1 Animals and diet

**Fig. 1:**
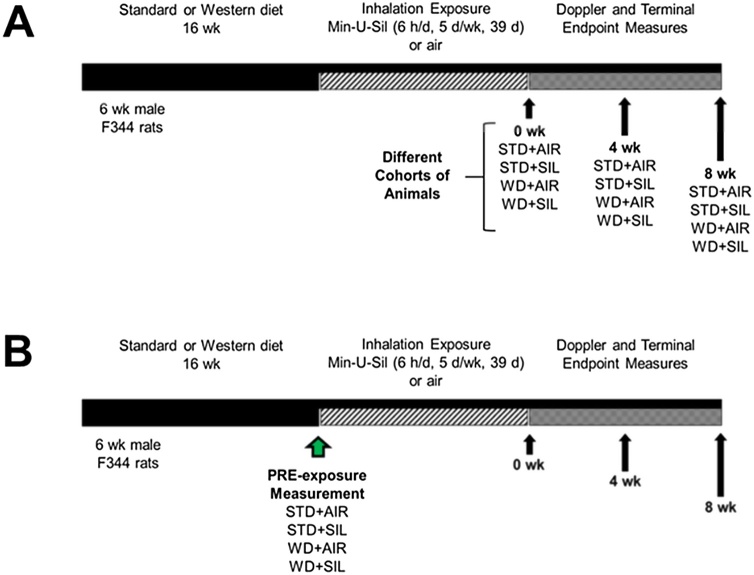
Experimental design for HFWD-induction of MetDys, silica inhalation exposure and endpoint experiments. A) Describes the design for single endpoint experiments using separate cohorts of animals (n = 8 for each group). B) Describes the design for repeated measures of fasting glucose and LDF experiments.

Six-wk old male Fischer (CDF) rats (F344/DuCrl) obtained from Charles River Laboratories, Inc. (Wilmington, MA) were divided into two dietary groups and fed either a commercially available “Western” diet (high-fat Western diet, HFWD; 45 % fat Kcal, sucrose 22.2 % by weight) or a standard rat chow (standard diet, STD; fat 6.2 % by weight, sucrose-free) for 16 wk, prior to the commencement of silica inhalation exposures. After 16 wk, animals were exposed by whole-body inhalation to silica for 6 h/d, 5 d/wk, 39 d or filtered air (control) and handled identically. Animals were euthanized for anthropometric and metabolic measures at 0, 4, and 8 wk post-exposure. Animals designated for the 8-wk post-exposure studies were used for repeatedly-measured laser doppler flowmetry (LFD), to assess arterial blood flow and pulse, and fasting glucose levels (Fig. 1).

All studies were conducted in facilities accredited by AAALAC International, were approved by the Institutional Animal Care and Use Committee (protocol 18-011) and were in compliance with the Public Health Service Policy on Humane Care and Use of Laboratory Animals and the NIH Guide for the Care and Use of Laboratory Animals. All animals were free of viral pathogens, parasites, mycoplasm, *Heliobacter* and cilia-associated respiratory bacillus. Animals were acclimated for one week upon arrival and housed in ventilated micro-isolator units supplied with HEPA-filtered laminar flow air (Lab Products OneCage; Seaford, DE), with Teklad Sanichip and Teklad Diamond Dry cellulose bedding (or Shepherd Specialty Paper’s Alpha-Dri cellulose, Shepherd Specialty Papers; Watertown, TN). They were provided filtered tap water and autoclaved Teklad Global 18 % protein rodent diet (Harlan Teklad; Madison, WI) *ad libitum*. Rats were housed in pairs under controlled light cycle (12 h light/12 h dark) and temperature (22–25 °C) conditions.

### 2.2 Silica-inhalation exposure

The F344 rat model of inhaled crystalline silica-induced pulmonary inflammation and fibrosis was characterized earlier in studies by Porter et al. [29] and Castranova et al. [24]. Briefly, animals exposed to silica inhalation for 40 d developed rapidly-increasing pulmonary inflammation and damage consistent with the pulmonary responses reported in humans; these pulmonary responses continued to develop despite cessation of silica exposure and decrease in silica lung burden [29], thereby modelling the progressive nature of human silicosis. In our study, animals from both STD and HFWD-consuming groups were exposed to 15 mg/m^3^ x 6 h/d x 39 d crystalline silica, or filtered air (control groups), and endpoints were measured at 0, 4 and 8 wk post-exposure to silica. The 0 wk endpoint reveals effects that occur during active silica exposure, and 4 and 8 wk post-exposure are time points at which pulmonary inflammation and fibrosis continue to progress after exposure has ended, such as is the case in accelerated silicosis in humans.

Crystalline silica (Min-U-Sil 5®; Berkeley Springs, WV; “SIL”) was aerosolized using an automated exposure system [31] that delivered airborne particles with median aerodynamic diameter of 1.6 μm and geometric standard deviation of 1.6. Target silica concentration (15 ± 1 mg/m^3^) was monitored and controlled within the exposure chamber in real time and control animals were exposed to filtered air and handled identically (Fig. 1).

### 2.3 Anthropometric and metabolic measures

Animals were euthanized using an overdose of sodium pentobarbital (200–300 mg/kg, Vortech; Dearborn, MI) administered by intraperitoneal injection, followed by exsanguination. Weight, nose-to-tail length, and abdominal girth were measured, and body mass index (BMI) was calculated. Blood was collected from the vena cava in EDTA-treated collection tubes and cells were analyzed using a ProCyte Dx Hematology Analyzer (IDEXX Laboratories, Inc.; Norcross, GA). Blood used for serum samples was collected in BD Vacutainer™ Serum Separation Tubes, left at room temperature for 1.5 h, and centrifuged at 25,000 *g* for 20 min. Fresh serum was used for measurements of high-density lipoprotein (HDL) (#ab65390, Abcam, Cambridge, MA) and blood chemistry (Catalyst Dx Chemistry Analyzer, IDEXX Laboratories, Inc). Epididymal fat pads were removed and weighed. Serum samples were stored at -80 °C until used for ELISA and cytokine analysis. ELISAs were conducted following the manufacturers’ protocols: leptin (#MOB00B, R&D Systems, Minneapolis, MN) and adiponectin (#Acrp30, R&D Systems). Insulin was measured using the Ultra-Sensitive Rat Insulin Elisa Kit (#90060, Crystal Chem; Elk Grove Village, IL). Serum cytokines were measured using the MSD V-PLEX Proinflammatory Panel 2 (rat) kit and MESO QuickPlex SQ 120 (Meso Scale Diagnostics; Rockville, MD).

### 2.4 Repeated measures of vascular function and blood glucose

Repeated measurements of fasting glucose levels, and blood flow and arterial pulse by laser Doppler flowmetry (LDF) were made at pre-exposure, and at 0, 4, and 8 wk after exposure to silica. Animals were fasted for 12−15 hours overnight (water available *ad libitum*) and fasting blood glucose was measured the next morning. The rat tail was sterilized with alcohol wipe then warmed for 45 s using a heating pad. An automatic lancet (Freestyle Lancing Device, 28-gauge sterile lancet, Abbott Laboratories; Alpharetta, GA) was used to prick the tail approximately 0.5 in. from the tip to obtain a small droplet of blood and glucose was measured with a hand-held glucometer (Freestyle Freedom Lite Blood Glucose Meter, Abbott Laboratories). Food was returned, along with water, and was available *ad libitum*.

LDF, a peripheral non-invasive procedure, was used to measure changes in rat tail arterial blood flow and arterial pulse. These measures were made in unfasted animals placed in Broome-style restrainers and recorded over a 15-min period. Mean blood flow was calculated and analyzed. Time series analyses were performed on the blood flow data (fast Fourier transform analysis, FFT) and two primary peaks in blood flow were identified, at 0.88–1 and 0.2 – 0.4 Hz.

### 2.5 Statistical analysis

Data were analyzed using JMP version 13.2, and SAS version 9.4 (SAS Institute; Cary, NC). Variables were analyzed using three-way analyses of variance (diet × exposure × time). Relevant pair-wise comparisons were generated using Fisher’s LSD test. For cytokine analysis, all values below the lower limit of detection (LLOD) were replaced with the LLOD/sqrt(2). LDF data were analyzed using repeated measures ANOVA, and data from the time series were analyzed using 3-way ANOVAs. All differences were considered significant at P < 0.05.

## 3 Results

### 3.1 HFWD consumption increases body weight, BMI, girth and epididymal fat pad mass

**Fig. 2:**
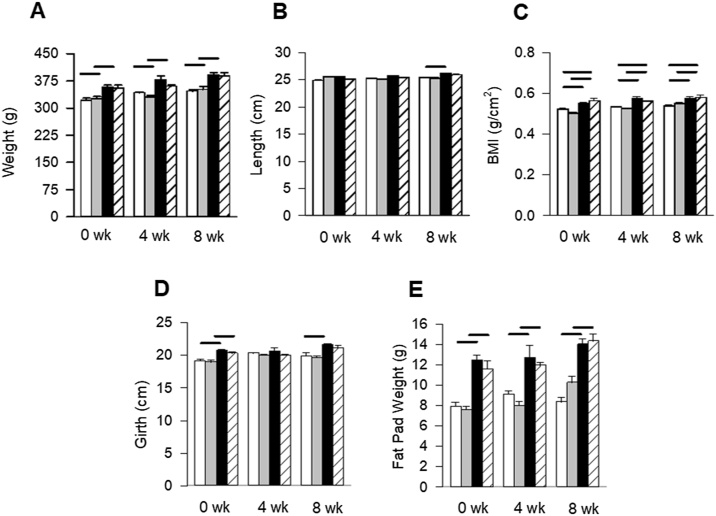
Graphs indicate measures of body weight (A), length (B), body mass index (BMI) (C), girth (D), and fat pad weight (E), at 0, 4 and 8 wk post-silica exposure. Consumption of a HFWD increased animal body and fat pad weight, and abdominal girth, at 0 and 8 wk. Silica exposure had no effect. Solid lines indicate significant differences between different exposure groups at a given time point. (P < 0.05; n = 6 – 8).

HFWD consumption increased body weight (12 %, 10 %, 13 %) (Fig. 2A), BMI (5.9 %, 7.7 %, 6.7 %) (Fig. 2C), abdominal girth (9%, 2%, 9%) (Fig. 2D), and epididymal fat pad weight (58 %, 40 %, 68 %) (Fig. 2E) compared to STD + AIR (P < 0.001); silica exposure had no effect on those parameters. Animal length was increased in the HFWD + AIR group compared to STD + AIR at 8 wk (Fig. 2B) but had no effect on the significance of BMI measures of those groups. Our results confirm that HFWD-consumption by F344 rats resulted in significant weight gain due to increased adipose tissue mass and that silica inhalation, regardless of diet, had no effect on these parameters.

### 3.2 Serum adipokines levels are altered by silica and HFWD

**Fig. 3:**
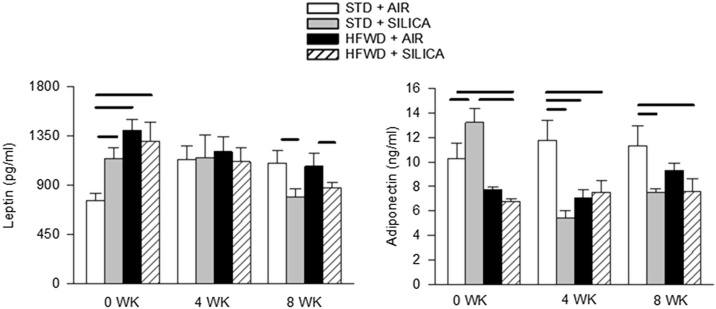
Leptin level (left panel) was increased at 0 wk by both silica and HFWD exposure at 0 wk but reduced by silica in both STD and HFWD groups at 8 wk. Adiponectin levels (right panel) were reduced by both silica and HFWD at all time points compared to STD exposure alone. These results indicate that both diet and silica exposure alter adipose tissue endocrine function. Solid lines indicate significant differences between different exposure groups at a given time point. (P < 0.05; n = 6 – 8 for each group).

Silica initially increased both leptin (Fig. 3, left panel) and adiponectin (Fig. 3, right panel) levels at 0 wk, but by 8 wk post-exposure both were significantly reduced by silica, regardless of the diet consumed and with no differences between STD + SIL and HFW + SIL groups. HFWD-consumption increased leptin and decreased adiponectin at 0 wk, but by 8 wk there was no longer a diet-induced effect.

### 3.3 HFWD consumption alters silica-induced serum lipids

**Fig. 4:**
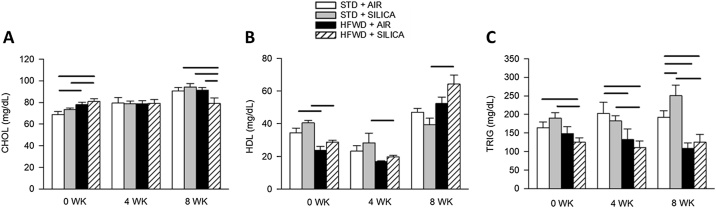
Effects of HFWD and silica inhalation on total cholesterol (A), HDL (B), and triglycerides (C) are shown. While HFWD initially increased cholesterol in both air and silica exposed groups at 0 wk compared to their STD controls, there were no differences at 4 wk, and levels decreased in the HFWD + SIL group by 8 wk compared to all other groups (A). HDL was reduced by HFWD in both air and silica groups at 0 wk but was only reduced by HFWD + SIL at 4 wk, followed by an increase in HDL by HFWD + SIL compared to STD + SIL (B). Triglycerides were reduced by HFWD in both exposure groups at all time points compared to their diet controls; at 8 wk triglycerides were significantly increased in the STD + SIL group compared to STD + AIR control (C). Solid lines indicate significant differences between different exposure groups at a given time point. (P < 0.05; n = 4 – 8).

Silica (STD + AIR) exposure has no effect on total cholesterol levels (Fig. 4A). HFWD-consumption increased total cholesterol levels in both silica- and air-exposed groups at 0 wk compared to STD controls. While there were no differences between the groups at 4 wk, at 8 wk cholesterol level in the HFWD + SIL was reduced compared to all other groups at that time point.

High-density lipoprotein (HDL) binds circulating lipids and carries them to the liver; HDL levels are typically decreased in MetDys [32]. Silica (STD + AIR) had no effect on HDL levels (Fig. 4B). HFWD-consumption reduced HDL levels at 0 wk compared STD + AIR and was without effect at other time points. In the combined exposure group (HFWD + SIL), HDL levels were reduced at 0 and 4 wk, and increased at 8 wk compared to STD + SIL.

Triglycerides (TRIGs) are created during food digestion but can also be released by the liver in response to carbohydrate intake [33]. Circulating TRIGs are typically elevated in MetDys. Silica increased TRIG at 8 wk (Fig. 4C). HFWD-consumption resulted in a decrease in TRIG levels at all time points compared to the respective STD-fed groups (P < 0.001). At 8 wk, combined HFWD + SIL reduced the silica-induced increase (STD + SIL) in TRIG.

### 3.4 HFWD and silica effects on fasting glucose and serum insulin

**Fig. 5:**
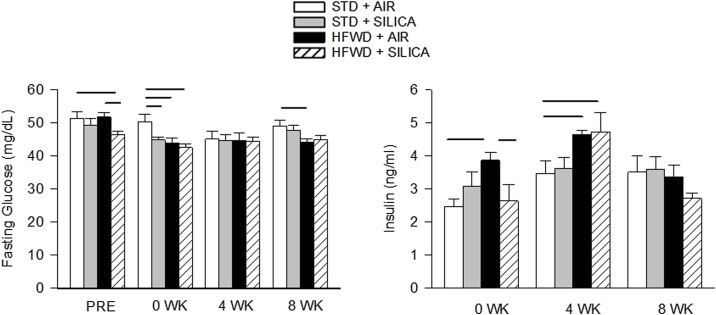
Effects of HFWD and silica inhalation on fasting glucose and non-fasting insulin. Fasting glucose (left panel) was reduced by silica reduced at 0 wk, and HFWD decreased fasting glucose levels at 0 and 8 wk compared to STD + AIR. Insulin levels (right panel) were increased by HFWD at 0 wk and 4 wk; HFWD + SIL reduced the insulin level at 0 wk compared to HFWD. Solid lines indicate significant differences between different exposure groups at a given time point. (P < 0.05; n = 7 – 8).

Insulin resistance is associated with elevated fasting blood glucose and hyperinsulemia. Repeated measures of fasting glucose were made in animals prior to the beginning of silica exposure (or air) (PRE), and at 0, 4 and 8 wk post-exposure (Fig. 5, left panel). Silica reduced fasting glucose level at 0 wk compared to STD + AIR. Fasting glucose levels were reduced by HFWD-consumption at 0 and 8 wk compared to STD + AIR. We found that fasting glucose level was significantly lower in the HFWD + SIL group prior to silica exposure (PRE), but this was a HFWD effect not a silica effect as the animals had not yet been exposed.

Non-fasting serum insulin was measured in the different cohorts of animals (Fig. 5, right panel). Silica had no effect on insulin levels. HFWD-consumption increased insulin at 0 and 4 wk compared to STD + AIR, and HFWD + SIL increased insulin compared to STD + AIR at 4 wk as well, but there were no differences between groups at 8 wk.

### 3.5 HFWD consumption impairs liver and kidney function

A full description of all the serum metabolites that were measured is included in Supplemental Table 1.

**Fig. 6:**
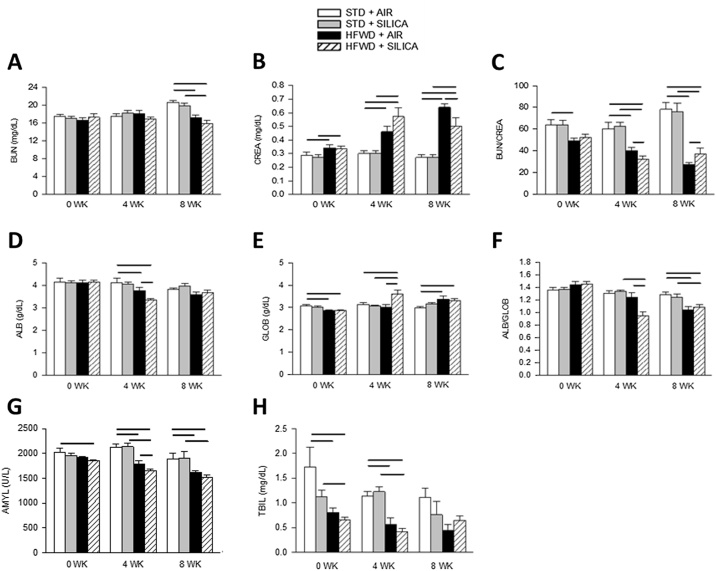
Consumption of HFWD alters blood chemistry panel. (A) Blood urea nitrogen (BUN) was decreased by HFWD-consumption at 8 wk, (B) creatine (CRE) was increased by HFWD-consumption at all time points, (C) BUN/CREA was decreased by HFWD in both air and silica exposed groups. (D) Albumin (ALB) was decreased by HFWD at 4 wk only, while (E) globulin (GLOB) was altered and (F) ALB/GLOB ratio was decreased by HFWD-consumption in both air and silica exposure groups. Solid lines indicate significant differences between different exposure groups at a given time point. (P < 0.05; n = 4 – 8).

A comprehensive blood panel was used to detect changes in liver, kidney, and pancreatic function. Blood urea nitrogen (BUN) is a waste product produced by the liver and filtered by the kidneys; reduced BUN level is an indicator of reduced liver function [34]. Silica (STD + SIL) had no effect on BUN level (Fig. 6A), but BUN was reduced by HFWD-consumption at 8 wk. Creatine (CREA), a product of muscle mass homeostasis, is filtered by the kidneys and serum concentrations typically do not fluctuate; increased CREA level is an indicator of reduced kidney filtration [34]. Silica had no effect on CREA (Fig. 6B). We found that HFWD consumption (HFWD + AIR) increased creatine levels at all time points, indicating a change in kidney function. BUN/CREA ratio is another measure of liver and kidney function, and reduced BUN/CREA ration is indicative of liver and kidney impairment [35]. Silica had no effect on BUN/CREA (Fig. 6C). HFWD-consumption reduced the BUN/CREA ratio compared to STD + AIR at all time points and was below the normal rat value of 70 (Supplemental Table 1). Levels of BUN, CREA and BUN/CREA in the HFWD + SIL group were not different compared to than HFWD + AIR, indicating those changes were strictly a diet-induced effect.

Albumin (ALB), a serum protein, and globulin (GLOB), an immunoglobulin, are produced by the liver and excreted by the kidneys. Changes in total protein levels, the level of either protein individually, or the ratio of ALB/GLOB [36], is used clinically to diagnose impairment of liver and kidney function and a reduction of albumin is associated with inflammation [36]. Silica (STD + SIL) had no effect on ALB, GLOB or ALB/GLOB levels (Fig. 6D, E, F). HFWD-consumption (HFWD + AIR) decreased ALB level at 4 wk compared to STD + AIR; GLOB levels were reduced at 0 wk and increased at 8 wk by HFWD (HFWD + AIR) compared to STD + AIR controls; and the ALB/GLOB ratio was reduced by HFWD at 4 and 8 wk compared to STD + AIR. HFWD + SIL was not different from HFWD + AIR groups except for GLOB at 4 wk, where GLOB was greater than all other groups at that time point. HFWD + SIL’s effects were similar to that of the HFWD groups and different compared to STD + AIR. The only difference between HFWD + SIL and HFWD + AIR occurred at 4 wk, when HFWD + SIL caused a greater decrease in ALB and ALB/GLOB compared to HFWD + AIR (Fig. 6D, F) and GLOB levels were increased in the HFWD + SIL group to a greater degree than all other groups (Fig. 6E). These results indicate that HFWD-consumption impairs liver and kidney function, and that HFWD + SIL induced further impairment at 4 wk that appeared to resolve back to HFWD levels by 8 wk.

Serum amylase (AMYL) is an enzyme produced by the pancreas, and we measured AMYL to determine changes in pancreatic function (Fig. 6G). Silica had no effect on serum AMYL levels. HFWD consumption (HFWD + AIR) reduced AMYL levels at 4 and 8 wk compared to STD + AIR. HFWD + SIL reduced AMYL levels at all time points compared to STD + AIR and was less than STD + SIL at 4 and 8 wk, and less than HFWD + AIR at 4 wk. Low serum amylase is associated with pancreatitis and impaired kidney function [37]. Our results indicate that HFWD-consumption may impair pancreatic function in addition to kidney function, and the reductions in AMYL of the HFWD + SIL groups can be attributed to the diet-induced effects.

Total bilirubin (TBIL) is a by-product produced by the breakdown of red blood cells and is produced in the liver; elevated serum TBIL is associated with lower risk of chronic kidney and cardiovascular disease in adults [38] (Fig. 6H). Silica had no effect on TBIL levels. HFWD consumption (HFWD + AIR) reduced TBIL levels at 0 and 4 wk compared to STD + AIR controls, and HFWD + SIL reduced TBIL at 0 and 4 wk compared with STD + SIL.

### 3.6 HFWD alters silica-induced complete blood count

**Fig. 7:**
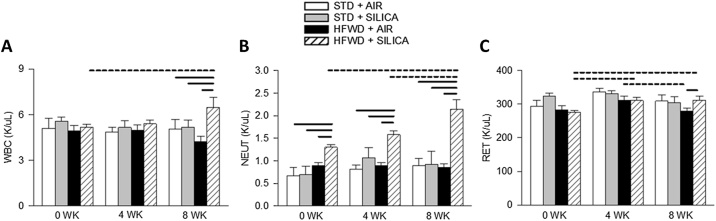
Combined exposure HFWD + SIL increased (A) white blood cells (WBC) at 8 wk compared to all groups and WBCs within the HFWD + SIL group over time. (B) Neutrophils (NEUT) levels were increased in the HFWD + SIL group compared to all other groups at all time points and within the HFWD + SIL group over time. (C) Reticulocytes (RET) were elevated at 4 wk within the HFWD + AIR group compared to 0 and 8 wk, while RETs increased over time within the HFWD + SIL group. Solid lines indicate significant differences between different exposure groups at a given time point; dashed lines indicate significant differences within a single exposure group compared at different time points. (P < 0.05; n = 4 – 8).

Complete blood count (CBC), and determination of blood cell differentials, are used as indicators of disease and/or infection. We assessed CBCs and cell differentials to determine the effects of HFWD consumption, silica, and combined exposure effects. An increase in white blood cells (WBC) is associated with infection. Silica (STD + AIR) and HFWD (HFWD + AIR) had no effect on systemic white blood cell levels (WBC) (Fig. 7A). HFWD + SIL, however, increased WBCs at 8 wk compared to all other groups and increased WBCs within the HFWD + SIL group between 0 and 8 wk (Fig. 7A).

Neutrophils (NEUT), a granulocytic type of WBC, becomes elevated in response to injury or infection. We found that neither silica (STD + AIR) nor HFWD (HFWD + AIR) had any effect on blood neutrophil (NEUT) levels (Fig. 7B). HFWD + SIL, however, increased neutrophils at 0, 4, and 8 wk compared to all other groups and also increased over time from 0 to 8 wk within the HFWD + SIL group (Fig. 7B).

Reticulocytes (RET), immature red blood cells, are measured to determine if the bone marrow is producing sufficient erythrocytes and can be used to detect anemia and bone marrow disorders [39]. Reticulocytes might also be increased in response to tissue hypoxia [40], as is found in silicotic patients. Blood reticulocytes concentrations were not altered by silica (STD + AIR) (Fig. 7C). Reticulocyte levels were reduced in the HFWD-fed group (HFWD + AIR) between 4 and 8 wk, but HFWD + SIL increased reticulocytes from 0 to 4 wk, and this level was maintained through 8 wk (Fig. 7C). There were no differences between the groups, except between HFWD + AIR and HFWD + SIL at 8 wk.

Levels of red blood cells, eosinophils, lymphocytes, and basophils were also measured, but there were no significant differences between the groups at any time points (Supplemental Fig. 1). At 0 wk, monocytes were increased in number in the STD + SIL group compared to the air-exposed STD and HFWD groups; at 8 wk in the HFWD + AIR group there was a significant reduction in monocyte level compared to the HFWD + SIL exposure group.

### 3.7 HFWD increases serum pro-inflammatory cytokines and alters silica-induced cytokines

**Fig. 8:**
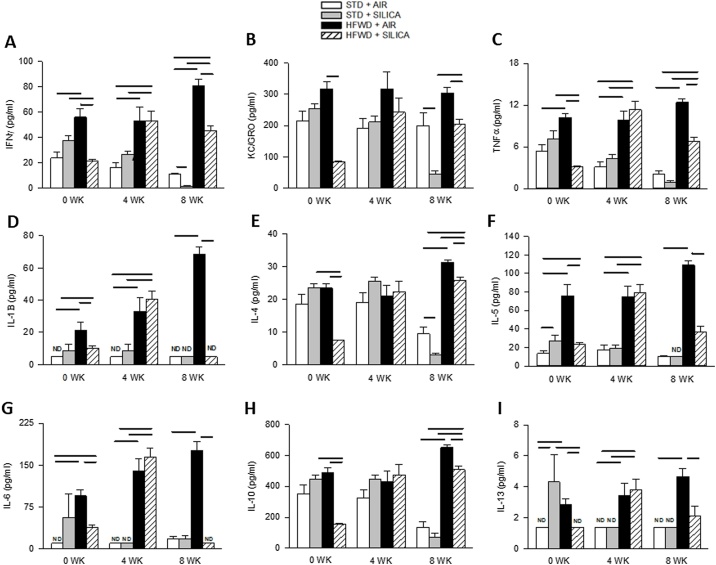
Effects of HFWD and silica inhalation on serum proinflammatory cytokines: (A) IFNγ, (B) KC/GRO, (C) TNFα, (D) IL-1β, (E) IL-4, (F) IL-5, (G) IL-6, (H) IL-10, and (I) IL-13. Silica exposure (STD + SIL) had little effect on systemic cytokines while HFWD consumption increased systemic cytokines at all time points (A-I). At 8 wk, HFWD + SIL reduced all HFWD-induced cytokines (A – I). ND indicates cytokine levels below LLOD. [LLODs (pg/mL): IFNγ = 0.65; IL-1β = 6.92; IL-4 = 0.69; IL-5 = 14.10; IL-6 = 13.80; KC/GRO = 1.04; TNFα = 0.72]. (P < 0.05; n = 8).

We examined serum pro-inflammatory cytokines as biomarkers of systemic inflammation resulting from diet, silica exposure, or synergistic interactions of the combined HFWD and silica exposures (Fig. 8). Silica exposure (STD + SIL) had little effect upon serum cytokines; but silica did increase IL-5 (Fig. 8F) and IL-13 (Fig. 8I) at 0 wk and decreased IFNγ (Fig. 8A) and KC/GRO (Fig. 8B) at 8 wk compared to STD + AIR cohorts. Consumption of a HFWD diet increased levels of all pro-inflammatory cytokines (Fig. 8A-I) compared to the STD + AIR group, with the most significant differences occurring by 8 wk. HFWD + SIL’s effects were complicated and varied over the different time points. At 0 wk cytokines IFNγ (Fig. 8A), KC/GRO (Fig. 8B), TNFα (Fig. 8C), IL-10 (Fig. 8H), and IL-13 (Fig. 8I) were reduced in amount by HFWD + SIL compared to STD + SIL. At 4 wk HFWD + SIL increased IFNγ (Fig. 8A), TNFα (Fig. 8C), IL-1B (Fig. 8D), IL-5 (Fig. 8F), IL-6 (Fig. 8G), IL-10 (Fig. 8H), IL-13 (Fig. 8I) were greater than STD + SIL. At 8 wk, HFWD + SIL increased IFNγ (Fig. 8A), KC/GRO (Fig. 8B), TNFα (Fig. 8C), IL-4 (Fig. 8E), and IL-10 (Fig. 8H) compared to STD + SIL. Overall, we found that HFWD-consumption altered silica-induced serum cytokines compared to STD + SIL controls.

### 3.8 HFWD and silica induce changes in arterial function

**Fig. 9:**
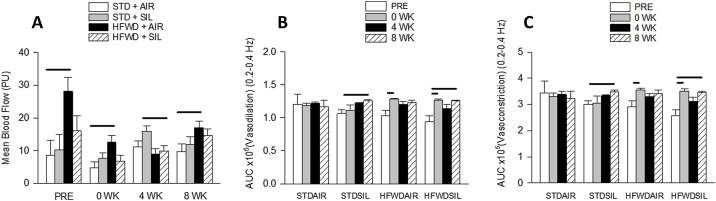
Effects of HFWD and silica inhalation on changes in tail artery function measured by the LDF technique. (A) HFWD + AIR increased mean blood flow compared to STD + AIR control at all time points. Changes in vasodilation (B) and vasoconstriction (C) within groups are shown over time. Silica increased vasodilation and vasoconstriction over time from pre-exposure to 8 wk in both STD and HFWD groups. HFWD increased arterial vasodilation and constriction from pre-exposure to 0 wk in both air and silica exposed groups. Combined HFWD + SIL had a cumulative effect on arterial dilation and constriction at both 0 and 8 wk compared to pre-exposure. Solid lines indicate significant differences between different exposure groups at a given time point. (P < 0.05; n = 8).

The LDF technique was employed to detect changes in rat tail artery mean blood flow and arterial pulse (Fig. 9). Consumption of a HFWD increased mean blood flow at the pre-inhalation, 0, and 8 wk repeatedly-measured time points compared to the STD + AIR control group (Fig. 9A). At 4 wk there was an increase in mean blood flow in the STD + SIL group compared to the HFWD + SIL cohort, but this effect was transient and not present at 8 wk post-exposure.

Changes in systemic vasodilation (Fig. 9B) and vasoconstriction (Fig. 9C) were determined by analysis of the area under the curve (AUC) at 0.2 – 0.4 Hz. A large AUC suggests the heart is beating stronger and a change in area is generally indicative of a change in blood pressure [41]. Silica-exposure resulted in an increase in vasodilation and vasoconstriction at 8 wk post-exposure in both the STD- and HFWD-fed groups compared to their pre-inhalation values. HFWD consumption resulted in an increase in the frequencies of vasodilation and vasoconstriction at 0 wk compared to the values before silica or air inhalation. Combined exposure of HFWD + SIL resulted in significant increases in vasodilation and vasoconstriction at both 0 and 8 wk post-exposure times compared to pre-inhalation values. Changes in arterial pulse were compared over time within each exposure group as there were no significant differences in arterial pulse between the four exposure groups at any time point. Our results indicate that consumption of a HFWD increased both mean blood flow at all time points and arterial pulse transiently; in the combined exposure HFWD + SIL group there was no change in mean blood flow but there were additive effects of increased arterial pulse at 0 and 8 wk.

## 4 Discussion

The three aims of this investigation were to: 1) determine the metabolic effects of HFWD consumption in the F344 rat; 2) define the metabolic effects of silica inhalation; and 3) ascertain whether metabolic effects resulting from silica inhalation are altered by HFWD-consumption. The most significant findings were: 1) silica inhalation reduced serum leptin and adiponectin levels, without effects on body or epididymal fat pad weight; 2) HFWD-consumption altered pro-inflammatory cytokines in silica-exposed animals, and 3) silica altered arterial pulse frequency and HFWD increased mean blood flow; vascular changes were additive in the combined exposure HFWD + SIL group. We observed that HFWD but not silica caused appreciable metabolic alterations and, surprisingly, that many of these alterations were blunted by silica exposure.

MetDys was confirmed by anthropometric measures. Consumption of the HFWD caused body weight gain, and increased BMI, abdominal girth and epididymal fat pad mass. Adipokines leptin and adiponectin were altered by both HFWD and silica exposure. While HFWD-induced adipokine dysregulation at 0 and 4 wk, there was no effect of HFWD on either adipokine at 8 wk. Silica exposure, on the other hand, altered serum adipokine levels at all time points, significantly reducing adiponectin and leptin levels regardless of diet at 8 wk. Adiponectin and adiponectin mRNA are reduced in obesity-induced adipose hypoxia [42]. Adipose hypoxia occurs due to oxygen’s limited diffusion potential within the AT [43], and as a result of increased of AT cell size and cell number [44]. Roberts et al. [45] reported a correlation between fat cell size and decreased serum leptin in rats fed a high-fat high-sucrose diet (HFHS). Opposing effects by HFWD and silica exposure suggest that these changes in adipokines result from two different mechanisms.

The NLRP3 inflammasome, a multiprotein oligomer, plays a significant role in the initiation of innate immune responses through release of pro-inflammatory cytokines [46,47]. In silicosis, the NLRP3 inflammasome is activated in AMs by phagocytosis of silica particles [27]; in obesity the NLRP3 inflammasome can be activated by factors released by stressed adipocytes and through activation of pathogen recognition receptors (PRRs) of AT macrophages [3]. IL-1β release is a hallmark of inflammasome activation. While activation of the NLRPS inflammasome is activated by silica in the lung, we did not find an increase of serum IL-1β in the STD + SIL groups, indicating that any inflammation in the lung remained largely compartmentalized. HFWD-consumption increased serum IL-1β along with the other pro-inflammatory cytokines, indicating the initiation of a systemic inflammatory response. Of greatest interest was the decrease in cytokines at 8 wk in the STD + SIL and HFWD + SIL groups compared to their respective diet-air controls, including reduction of IL-1β to an undetectable level in the HFWD + SIL group. There was no reduction in body and epididymal fat pad weight in the silica-exposed groups compared to their diet controls, and, therefore, this reduction in cytokines was not caused by a decrease in AT mass. While many immune cells including AMs, monocytes, T and B cells infiltrate AT in obesity, the highly significant decreases in IL-1β and IL-6 suggest that inhibition of the inflammasome in AT macrophages could play a pivotal role in the reduction of HFWD-induced cytokines. Nemmar et al. [48] reported that intratracheally-instilled ultrafine nanocolloid albumin particles diffused from the lungs into the liver, spleen, kidneys, and brain of hamsters. Inhaled ultrafine crystalline silica particles [49] may also migrate from the lungs to AT macrophages through the blood and inhibit cytokine production and increase antioxidant resolution pathways. The presence of silica particles in AT is under investigation.

AT dysfunction is associated with oxidative stress. Roberts et al. [50] found that the levels of plasma malondialdehyde, a marker of lipid peroxidation by ROS, and nitrotyrosine, a biproduct of oxidative stress, were increased in HFHS-fed rats. Oxidative stress likely played a role in our study as HFWD-induced changes in liver, kidney and mean blood flow were observed. Conversely, antioxidant defenses may have played a role in the reduction systemic inflammation in silica-exposed animals. Leonard et al. [51] discovered an increase in bronchoalveolar catalase, an antioxidant enzyme, and total antioxidant status in serum of their murine asbestosis model. Pulmonary inflammation and ROS increased following asbestos exposure, blood antioxidant activity increased as well; however, systemic inflammatory cytokine levels were reduced. Increased serum antioxidant and BAL catalase levels correlated with reduced clearance rate of TEMPOL, a measurable serum radical. We did not measure catalase activity; however, reticulocytes increased significantly over time within the HFWD + SIL group. Reticulocytes, immature erythrocytes, contain high catalase enzyme activity [52]. Increased numbers of reticulocytes in the HFWD + SIL group also suggest a possibly increased antioxidant response.

Less understood is silica’s role in immune modulation and association with autoimmune diseases. Silicosis patients are at increased risk for the development of autoimmune diseases, including systemic lupus erythematosus, systemic sclerosis, rheumatoid arthritis [30], and increased susceptibility to tuberculosis [53]. Zhao et al. [54] found that silica exposure up-regulated over 500 genes associated with immune responses, extracellular matrix remodeling, cell adhesion and migration, signaling pathways and various regulation processes of the lung, yet there is little known regarding the mechanisms of silica-induced autoimmune disease. In this study we found that silica exposure reduced systemic pro-inflammatory cytokines and adipokines, yet there was no decrease in AT mass. Silica’s adjuvant properties, resulting in increased production of autoimmune related antibodies, is documented [55]. It is plausible that the silica-induced reduction of systemic cytokines and adipokines could be a result of an autoimmune response targeting the adipose tissue, but further investigation of the alteration of silica-induced adipose immune status and toxicity are required.

We observed hyperemia, i.e., increased blood flow, in animals consuming a HFWD. Hyperemia is a functional dilation of the arteries that accommodates the increased need for blood flow during exercise, but dysfunctional hyperemia is associated with obesity in humans. Obesity-associated hyperemia are related to endothelial dysfunction induced by increased ROS, decreased nitric oxide synthase (NOS), and hyperglycemia [56]. We observed increased arterial pulse (vasodilation and vasoconstriction) in animals fed the HFWD leptin, and in animals exposed to silica, which correlated with changes in adiponectin and leptin. Abnormal and altered adipokine levels have been shown to play a role in the etiology of cardiovascular disease [57]. Leptin regulates blood pressure via sympathetic activation, vasodilation of blood vessels and stimulation of natriuresis in the kidney [58], and increases neuronal NOS (nNOS) but not endothelial NOS (eNOS) [35]. Adiponectin induces phosphorylation of AMPKα, activation of adenosine monophosphate (AMPK) pathways. and NOS activity [59,60]. Li et al. [60] demonstrated that animals fed a high fat diet responded with increased serum leptin levels, decreased adiponectin and NO levels, and increased AMPKα expression in endothelial tissue. After treatment with AICAR, an AMPK activator, adiponectin and NO levels were restored, and leptin levels decreased. Another study found that aortic rings of adiponectin^−/−^ mice have greater concentrations of superoxide anion and peroxynitrite, and this effect was reversed with recombinant adiponectin [61]. We cannot pinpoint the exact contributions of changes in leptin vs. adiponectin in the arterial pulse changes that we found; however, the effects of HFWD-consumption and silica-exposure were additive in the HFWD + SIL group and this indicates that those effects arise from different mechanisms.

Surprisingly, none of the animals this study developed insulin resistance. Fasting glucose was decreased by HFWD while insulin increased at 0 and 4 wk, and was not different from other groups at 8 wk. Our results differed from a long-term study in which *female* Fischer rats fed a high-fat high- simple carbohydrate (HFHS) diet developed insulin resistance [62,63]. Interestingly, blood glucose levels were not a significant predictor of lung injury in the first responders at the World Trade Center [11].

HFWD consumption reduced triglyceride levels but produced variable and moderate effects on total cholesterol and HDL levels. Antonini et al. [64,16] reported that Fisher rats fed a HFWD had variable susceptibility to hypertriglyceremia. In our study, silica exposure alone significantly increased triglyceride level by 8 wk, and HFWD + SIL reduced total cholesterol level and increased HDL level at 8 wk compared to the STD diet controls. We conclude that silica inhalation alters lipid profiles, at least in our animal model, but further investigation is required to determine if silica exposure promotes hepatic lipidosis.

A metabolic blood panel was used to access the overall health of the animals. HFWD-fed groups exhibited changes in BUN, CREA, and BUN/CREA, indicating changes in liver and kidney function; reduction in serum amylase levels indicated decreased pancreatic function. HFWD consumption is associated with accumulation of lipids in the liver [16,65] and kidney [16,66], and inflammation and enhanced oxidative damage of the pancreas, heart, spleen, lung, intestine, liver, and kidney [67]. Interestingly, we found that HFWD + SIL reduced many of silica-induced serum metabolites by 8 wk, similar to observations that fine particulate matter exposure stimulated hepatic autophagy in mice maintained on a high-fat diet, attenuating lipid accumulation in the liver [68].

### 4.1 Limitations and future directions

There are limitations in the translation of our animal study to human disease. HFWD consumption induced-MetDys, evidenced by weight gain, altered adipokines and changes in vascular function; however, hypercholesterolemia or insulin resistance were not observed. Obesity is associated with increased risk of nonalcoholic fatty liver disease (NAFLD) and steatosis, with changes in liver function appearing prior to changes in serum lipid profiles [69]. Only serum lipid profiles were measured in this study, and, therefore, additional investigation of liver morphology and hepatic function endpoints would give a more complete picture of HFWD and HFWD + SIL-induced effects in the liver. Fischer rats, an inbred strain, have been used in MetDys studies [16,62,64,63,45] and occupational hazard studies [16,24,28]; however, induction of all conditions associated with human MetDys is difficult to achieve in the rat. Genetic obesity models, including leptin-deficient fatty (*fa/fa)* Zucker rats or (*ob/ob*) mice, are used to investigate obesity and metabolic dysfunction [70], yet these genetic models of obesity do not include changes in leptin, and, therefore, cannot completely mimic the diet-induced human disease. Human MetDys is difficult to reproduce in its entirety in animals due to complex multifactorial combinations of genetic and environmental factors [71,72]. Nonetheless, future studies including measurement of fasting serum insulin levels, and the calculation of HOMA-IR (homeostatic model assessment for insulin resistance) to approximate insulin resistance and HOMA-B (homeostatic model assessment of β-cell function), an index of insulin secretory function, would provide further insight into silica’s effects on insulin production and β-cell toxicity. Secondly, food consumption was not measured; however, there was no indication of decreased food consumption in the silica treatment groups. Body and fat pad weight were consistent compared to air controls, and, therefore, is not a likely explanation for the reduction in adipokines or cytokines levels in silica- exposed animals at 8 wk. Thirdly, oxidative stress plays a significant role in obesity-induced AT inflammation and silica-induced pulmonary inflammation. We did not measure serum antioxidant activity; therefore, we can only speculate about the contribution of anti-oxidative responses to the reduction of HFWD-induced cytokines by silica. Silica-induced alteration of serum adipokines and serum cytokines indicate possible adipose toxicity and further investigation of adipose morphology, adipokine gene expression, and NLRP3 activation endpoints are required to disentangle this complex observation. Lastly, our study used “aged” silica, not freshly fractured particles to which workers are exposed and which causes greater pulmonary inflammation [73].

### 4.2 Conclusions

In summary, we found that HFWD-consumption alters silica-induced metabolic responses, silica-inhalation has adipose endocrine function disrupting effects, and both HFWD and silica, alone and in combination, alter arterial function in the F344 rat. Our results suggest an increased susceptibility to silica-induced metabolic disorder in the presence of HFWD-consumption. These previously unknown interactions between diet, occupational silica exposure, and adipose function are significant and warrant further investigation.

## Data set

The original data are available at https://www.cdc.gov/niosh/data/datasets/rd-1019-2021-0/.

## Disclaimer

The findings and conclusions in this report are those of the author(s) and do not necessarily represent the official position of the National Institute for Occupational Safety and Health, Centers for Disease Control and Prevention.

## Funding

Funding was provided by the 10.13039/100000125National Institute for Occupational Safety and Health, Project Number 9390DT3.

## Declaration of Competing Interest

The authors report no declarations of interest.

## Figures and Tables

**Figure:**