# Supplementary material for: High-fat western diet-consumption alters crystalline silica-induced serum adipokines, inflammatory cytokines and arterial blood flow in the F344 rat
Source: Toxicol Rep. 2021 Dec 7;9:12–21. doi: 10.1016/j.toxrep.2021.12.001 (PMC8683385; doi:10.1016/j.toxrep.2021.12.001)
Supplement: Supplementary file 1 [file mmc1.docx]

**Supplemental Methods Section**

**Animals**

Five-week old Male Fischer F344/DuCrl rats (CDF) were purchased from Charles River Laboratories (Raleigh, NC), housed in pairs and acclimated for one week in cages (Lab Products OneCage 2100 rat cages; Lab Products, Inc., Seaford, DE) containing a combination of Sanichip (Envigo) and Alpha Dri bedding (Shepherd Specialty Papers). Water and standard rat chow (2018, Envigo) were available ad libitum and animals were kept on a 12-h light – 12-h dark schedule. These studies were approved by the Institutional Animal Care and Use Committee and conducted in facilities fully accredited by the Association for the Assessment and Accreditation of Laboratory Animal Care International.

**Diet**

All animals were fed a standard rat chow upon arrival and first week of acclimation. After acclimation, animals were randomly divided and fed either a commercially available 45% fat “Western” diet (HFWD)[Teklad Cutom Diet, TD.06415, Envigo; fat 45% Kcal (lard 19.5%, soybean oil 3% by weight), carbohydrate 36.2% Kcal (sucrose 22.2% by weight), protein 19% Kcal] or a standard rat chow (STD)[Teklad 2018, Envigo; fat 6.2% by weight, carbohydrate 45% by weight (whole grain sources), protein 18.0% by weight, fiber 4% by weight]. Fatty acid profile of the HFWD contains 36% saturated, 47% monosaturated, and 17% polyunsaturated fat. Animals were maintained on their assigned diets throughout the study.

**Silica Inhalation Exposure**

A cohort of 96 rats were divided into two groups containing 48 rats and fed either a HFWD or STD for 16 wk as previously described. These groups were then subdivided into silica-exposed or filtered air (control) groups (24 rats per group). Rats were exposed by inhalation to silica (Min-U-Sil 5, 15 mg/m^3^) or filtered air (control group) for 6 h per d, 5 days per week, for 39 days. Food and water were not available during the exposure period, which occurred during the 12-hour light cycle. After the completion of exposure, the animals were divided into 0-, 4- and 8-week post-exposure groups (8 rats per group) and housed under normal conditions until use. The 8-week post-exposure silica and control animals were used for repeated measures of weight gain, fasting glucose and laser doppler at the pre- and post-inhalation exposure time points. All rats were maintained on their assigned diet for the entirety of the study (Fig 1).

**Silica Exposure Chamber**

Min-U-Sil® 5 crystalline silica (US Silica; Berkeley Springs, WV) was used in generating an aerosol using a previously described system (McKinney et. al. 2013).  The system was an automated computer-controlled exposure system that was used to deliver precise concentrations of uniformly dispersed airborne silica particles with a size distribution within the respirable range.  In brief, the system used an acoustical particle generator with a venturi stage to de-agglomerate the particles.  The mass-median aerodynamic diameter of the airborne silica particles within the exposure chamber was 1.6 µm with a geometric standard deviation of 1.6.   The target silica concentration (15 ± 1 mg/m^3^) in the exposure chamber were monitored and controlled in real time automatically by the exposure system.  The only change from this previously described system was the installation of a larger exposure chamber capable of exposing more animals.

# **Anthropometric Measures, Metabolic Panel, Complete Blood Count (CBC)**

At the designated silica post-exposure time points, rats were euthanized with an overdose of pentobarbital (200-300 mg/kg) and terminal endpoint measurements were taken. Animal weight, length (nose to base of tail), and abdominal girth were measured. Body to mass index (BMI) was calculated for each animal as weight (g)/length^2^ (cm^2^). A vertical surgical incision was made opening the abdominal cavity and blood was drawn via the descending vena cava. Whole blood samples used for differential blood cell count (CBC) were collected in EDTA-treated collection tubes to prevent coagulation (Vet Collect Tubes, #98-11073-01, IDEXX Laboratories, Inc.) measured by ProCyte Dx Hematology Analyzer (IDEXX Laboratories, Inc., Westbrook, ME). Blood to be used for serum samples were collected in BD Vacutainer™ SST™ Serum Separation Tubes, left at room temperature for 1.5 hour and centrifuged 25,000 g for 20 min. Serum was used for measurements of high-density lipoprotein (HDL)(ab65390; Abcam, Cambridge, MA), blood chemistry (Catalyst Dx Chemistry Analyzer; IDEXX Laboratories, Inc.), adipokine (leptin and adiponectin), insulin, and serum cytokine analysis. Bronchoalveolar lavage (BAL) fluid was collected from the left lung. The right lung was clamped at the right bronchus using a small hemostat. Next, a small vertical incision was made in the trachea to allow insertion of an 18-gauge needle and the needle was secured with suture. A syringe containing ice-cold PBS was attached to the needle and the lung was inflated with 5 ml of cold PBS, withdrawn, and retained on ice for BAL cytokine analysis. Epididymal fat pads were removed and weighed. Serum and BAL samples were stored at -80˚ C until used for ELISA and cytokine analysis.

# **Leptin, Adiponectin, and Insulin ELISAs**

ELISA assays were conducted using the manufacturer protocols and plates were analyzed using a Varioskan LUX Multimode Microplate Reader (software SkanIt version 4.1; Thermo Fisher Scientific, Inc., Waltham, MA). Serum samples for use in leptin assay (#MOB00B, R&D Systems; Minneapolis, MN) were diluted 2-fold; serum samples for adiponectin (#Acrp30, R&D Systems)were diluted 100,000-fold; serum samples for insulin (Ultra-Sensitive Rat Insulin Elisa Kit (#90060; Crystal Chem, Elk Grove Village, IL) were not diluted and the “wide range assay” protocol was used.

# **Cytokine Analysis**

# Serum samples were stored at -80˚ C until used for cytokine analysis. Serum cytokines were measured using the MSD V-PLEX Proinflammatory Panel 2 (rat) kit following manufacturer’s protocol and analyzed using the MESO QuickPlex SQ 120 (Meso Scale Diagnostics, Rockville, MD

# **Repeated Measures**

Animals designated for 8 wk terminal measures were used for repeated measure experiments at pre-exposure, 0, 4, 8 wk post-exposure to silica or air (Fig. 1b). Repeated measures of fasting glucose, body mass, tail artery blood flow and function were made as described below.

# **Fasting glucose measurement**

At the designated time points, animals were fasted for 12-15 hours overnight (water available ad libitum) and fasting blood glucose was measured the next morning. The rat tail was sterilized with an alcohol wipe then warmed for 45 sec using a heating pad. An automatic lancet (Freestyle Lancing Device, 28-gauge sterile lancet; Abbott Laboratories, Alpharetta, GA) was used to prick the tail approximately 0.5 inches from the tip to obtain a small droplet of blood and glucose was measured with a hand-held glucometer (Freestyle Freedom Lite Blood Glucose Meter; Abbott Laboratories). Food was returned, along with water, and was available ad libitum for 24-h prior to flowmetry study at each time point.

# **Laser Doppler Flowmetry (LDF)**

Laser Doppler flowmetry measurement, also known as perfusion monitoring, is a non-invasive procedure used to determine changes in arterial blood flow of the rat tail. Light emitted at 780 nm via the laser probe scatters and is partially absorbed by the tissue (~0.5-1 mm depth). Light reaching the red blood cells (RBC) undergoes a change in frequency known as the Doppler Shift, while light reaching static tissue undergoes no change. These data are collected and converted to an electrical signal (Periscan PIM-2; Perimed Co. Ltd.) for analysis. The magnitude add frequency of the Doppler Shift is directly related to the number of RBC and velocity of blood flow at the site.  For this study rats were placed in a Broome-style restrainer with the tail placed over the Doppler laser, ~2 inches caudal to from the first tail vertebrate, and blood flow was recorded through the tail artery for a 15-minute period. An upper and lower threshold of 200 and 2 perfusion units (PU) was set, respectively.  Data that fell outside of the threshold levels (because of movement of the animals) were replaced using a running means method. This method involves using the 20 values before the out-of-range data and the 20 values after the out-of-range data to generate a number to replace the out-of-range value.  The smoothed data sets were then individually loaded into Jmp 13.0.0 (SAS Institute, Cary NC) and data were analyzed. Mean blood flow was calculated and analyzed; time series analysis was run on the 0.88 – 1 Hz and 0.2 – 0.4 Hz peaks. Statistical difference between groups was determined using repeated measures ANOVA.

**Statistics**

Data were analyzed using JMP version 13.2. Variables were analyzed using three-way analyses of variance (Diet by Treatment by Time). Relevant pairwise comparisons were generated using Fishers LSD test. LDF data was analyzed using Repeated Measures ANOVA. All differences were considered significant at p<0.05.
