# Supplementary material for: High-fat western diet-consumption alters crystalline silica-induced serum adipokines, inflammatory cytokines and arterial blood flow in the F344 rat
Source: Toxicol Rep. 2021 Dec 7;9:12–21. doi: 10.1016/j.toxrep.2021.12.001 (PMC8683385; doi:10.1016/j.toxrep.2021.12.001)
Supplement: Supplementary file 2 [file mmc2.docx]

**Supplemental Table 1.** Effects of HFWD and silica inhalation on the blood chemistry panel.

|  | **CREA** | **BUN** | **BUN/CREA** | **PHOS** | **CA** | **TP** | **ALB** | **GLOB** | **ALB/GLOB** | **ALT** | **ALKP** | **GGT** | **TBIL** | **CHOL** | **TRIG** | AMYL |
| --- | --- | --- | --- | --- | --- | --- | --- | --- | --- | --- | --- | --- | --- | --- | --- | --- |
| **Unit** | **(mg/dL)** | **(mg/dL)** | **(ratio)** | **(mg/dL)** | **(mg/dL)** | **(g/dL)** | **(g/dL)** | **(g/dL)** | **(ratio)** | **(U/L)** | **(U/L)** | **(U/L)** | **(mg/dL)** | **(mg/dL)** | **(mg/dL)** | **(U/L)** |
| **NORMAL RANGE** | **0.1 - 0.6** | **9 - 21** |  | **5.8 - 11.2** | **5.3 - 11.6** | **5.3 - 6.9** | **3.8 - 4.8** | **1.5 - 2.8** |  | **20 - 61** | **16 - 302** | **1 - 6** | **0.1 - 0.7** | **20 - 92** | **26 - 108** | **326 - 2246** |
|  |  |  |  |  |  |  |  |  |  |  |  |  |  |  |  |  |
| **STD AIR 0 wk** | 0.3 ± 0.0 | 17.5 ± 0.4 | 63.6 ± 5.3 | 10.1 ± 0.6 | 10.1 ± 0.2 | ***7.2 ± 0.2*** | 4.2 ± 0.2 | ***3.1 ± 0.1*** | 1.4 ± 0.1 | ***413 ± 91*** | 118 ± 11 | ***0.4 ± 0.4*** | ***1.7 ± 0.4*** | 68.8 ± 2.8 | ***164 ± 15*** | 2032 ± 72 |
| **STD SIL 0 wk** | 0.3 ± 0.0 | 17.0 ± 0.4 | 63.9 ± 4.4 | 10.6 ± 0.4 | 10.9 ± 0.4 | ***7.1 ± 0.1*** | 4.1 ± 0.1 | ***3.0 ± 0.0*** | 1.4 ± 0.0 | ***521 ± 67*** | 138 ± 7 | ***0.4 ± 0.4*** | ***1.1 ± 0.1*** | 73.3 ± 1.6 | ***190 ± 51*** | 1961 ± 40 |
| **HF AIR 0 wk** | 0.3 ± 0.0 | 16.6 ± 0.5 | 49.0 ± 2.9 | 9.8 ± 0.6 | 10.5 ± 0.2 | ***7.0 ± 0.1*** | 4.1 ± 0.1 | ***2.9 ± 0.0*** | 1.4 ± 0.1 | ***234 ± 43*** | 155 ± 7 | ***0.3 ± 0.3*** | 0.8 ± 0.1 | 78.0 ± 2.4 | ***148 ± 18*** | 1922 ± 18 |
| **HF SIL 0 wk** | 0.3 ± 0.0 | 17.3 ± 0.8 | 52.1 ± 3.0 | 9.9 ± 0.3 | 10.8 ± 0.2 | ***7.0 ± 0.1*** | 4.2 ± 0.1 | ***2.9 ± 0.0*** | 1.5 ± 0.0 | ***224 ± 31*** | 146 ± 8 | ***0.0 ± 0.0*** | 0.7 ± 0.0 | 81.0 ± 2.5 | ***125 ± 12*** | 1850 ± 25 |
|  |  |  |  |  |  |  |  |  |  |  |  |  |  |  |  |  |
| **STD AIR 4 wk** | 0.3 ± 0.0 | 18.6 ± 1.3 | 59.6 ± 5.1 | 15.2 ± 4.8 | 10.7 ± 0.5 | ***7.9 ± 0.7*** | 4.3 ± 0.3 | ***3.5 ± 0.4*** | 1.3 ± 0.1 | ***559 ± 51*** | 151 ± 17 | ***0.3 ± 0.3*** | ***1.4 ± 0.3*** | 81.5 ± 4.8 | ***205 ± 26*** | 2290 ± 173 |
| **STD SIL 4 wk** | 0.3 ± 0.0 | 18.3 ± 0.5 | 62.5 ± 3.9 | 10.1 ± 0.6 | 11.0 ± 0.4 | ***7.1 ± 0.1*** | 8.8 ± 4.7 | ***3.1 ± 0.0*** | 1.3 ± 0.0 | ***527 ± 51*** | 137 ± 6 | ***0.0 ± 0.0*** | ***1.2 ± 0.1*** | 78.9 ± 2.3 | ***182 ± 14*** | 2138 ± 72 |
| **HF AIR 4 wk** | 0.5 ± 0.0 | 18.0 ± 0.7 | 40.2 ± 2.9 | 9.7 ± 0.7 | 10.0 ± 0.2 | ***6.8 ± 0.1*** | 3.8 ± 0.1 | ***3.0 ± 0.1*** | 1.2 ± 0.1 | ***188 ± 56*** | 148 ± 12 | ***0.0 ± 0.0*** | 0.6 ± 0.1 | 78.8 ± 2.9 | ***133 ± 28*** | 1797 ± 60 |
| **HF SIL 4 wk** | 0.6 ± 0.1 | 16.9 ± 0.5 | 31.9 ± 3.5 | 8.5 ± 0.3 | 9.6 ± 0.1 | ***6.9 ± 0.1*** | 3.4 ± 0.1 | ***3.6 ± 0.2*** | 1.0 ± 0.1 | ***195 ± 45*** | 143 ± 8 | ***0.1 ± 0.1*** | 0.4 ± 0.1 | 79.1 ± 3.5 | ***111 ± 17*** | 1656 ± 39 |
|  |  |  |  |  |  |  |  |  |  |  |  |  |  |  |  |  |
| **STD AIR 8 wk** | 0.3 ± 0.0 | 20.6 ± 0.4 | 78.3 ± 6.4 | 10.3 ± 0.5 | 10.4 ± 0.2 | ***6.8 ± 0.1*** | 3.8 ± 0.1 | ***3.0 ± 0.1*** | 1.3 ± 0.0 | ***384 ± 76*** | 159 ± 9 | ***0.3 ± 0.2*** | ***1.1 ± 0.2*** | 90.7 ± 2.9 | ***193 ± 18*** | 1896 ± 117 |
| **STD SIL 8 wk** | 0.3 ± 0.0 | 19.9 ± 0.6 | 76.1 ± 7.6 | 10.8 ± 1.1 | 10.6 ± 0.3 | ***7.1 ± 0.1*** | 4.0 ± 0.1 | ***3.2 ± 0.1*** | 1.2 ± 0.1 | ***365 ± 78*** | 165 ± 21 | ***0.0 ± 0.0*** | ***1.2 ± 0.3*** | 94.1 ± 3.4 | ***251 ± 28*** | 1911 ± 123 |
| **HF AIR 8 wk** | 0.6 ± 0.0 | 17.2 ± 0.6 | 27.2 ± 1.8 | 8.6 ± 0.5 | 9.7 ± 0.1 | ***7.0 ± 0.2*** | 3.6 ± 0.1 | ***3.4 ± 0.1*** | 1.0 ± 0.1 | ***165 ± 72*** | 131 ± 13 | ***0.2 ± 0.2*** | 0.4 ± 0.1 | 91.2 ± 2.5 | ***109 ± 15*** | 1618 ± 42 |
| **HF SIL 8 wk** | 0.9 ± 0.4 | 15.8 ± 0.7 | 37.2 ± 5.4 | 9.0 ± 0.4 | 9.8 ± 0.1 | ***6.9 ± 0.1*** | 3.7 ± 0.1 | ***3.3 ± 0.1*** | 1.1 ± 0.0 | ***129 ± 26*** | 98 ± 4 | ***0.0 ± 0.0*** | 0.7 ± 0.1 | 79.0 ± 5.2 | ***125 ± 21*** | 1520 ± 47 |

The values shown in this table are means ± SEM.
